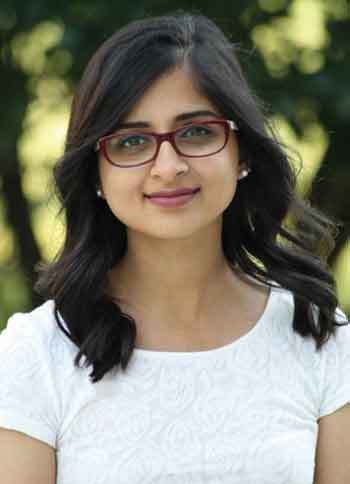# Participatory methods for Inuit public health promotion and programme evaluation in Nunatsiavut, Canada

**DOI:** 10.1080/22423982.2017.1343637

**Published:** 2017-07-09

**Authors:** Manpreet Saini

**Affiliations:** ^a^ Department of Population Medicine, University of Guelph, Guelph, Canada

**Keywords:** Participatory methods, Inuit, Nunatsiavut, whiteboard video, evaluations

## Abstract

Engaging stakeholders is crucial for health promotion and programme evaluations; understanding how to best engage stakeholders is less clear, especially within Indigenous communities. The objectives of this thesis research were to use participatory methods to: (1) co-develop and evaluate a whiteboard video for use as a public health promotion tool in Rigolet, Nunatsiavut, and (2) develop and validate a framework for participatory evaluation of Inuit public health initiatives in Nunatsiavut, Labrador. Data collection tools included interactive workshops, community events, interviews, focus-group discussions and surveys. Results indicated the whiteboard video was an engaging and suitable medium for sharing public health messaging due to its contextually relevant elements. Participants identified 4 foundational evaluation framework components necessary to conduct appropriate evaluations, including: (1) community engagement, (2) collaborative evaluation development, (3) tailored evaluation data collection and (4) evaluation scope. This research illustrates stakeholder participation is critical to develop and evaluate contextually relevant public health initiatives in Nunatsiavut, Labrador and should be considered in other Indigenous communities.